# Cardiac-related symptoms in individuals aged ≥65 years without diagnosed cardiac disease: insights from the NORSCREEN trial

**DOI:** 10.1093/ehjopen/oeag032

**Published:** 2026-02-20

**Authors:** Jarle Jortveit, Miroslav Boskovic, Marius B Haraldsen, Trygve Berge, Bjørnar L Grenne, John Munkhaugen, Sigrun Halvorsen

**Affiliations:** Department of Cardiology, Sørlandet Hospital HF, Arendal, Box 416, Lundsiden, Kristiansand 4604, Norway; Department of Cardiology, Sorlandet Hospital, Kristiansand, Box 416, Lundsiden, Kristiansand 4604, Norway; Faculty of Medicine, University of Oslo, PO Box 1078, Blindern, Oslo 0316, Norway; Faculty of Medicine, University of Oslo, PO Box 1078, Blindern, Oslo 0316, Norway; Department of Cardiology, Oslo University Hospital Ullevaal, PO Box 4950, Nydalen, Oslo 0424, Norway; Department of Medical Research, Baerum Hospital, Vestre Viken Hospital Trust, PO Box 800, Drammen 3004, Norway; Department of Internal Medicine, Baerum Hospital, Vestre Viken Hospital Trust, PO Box 800, Drammen 3004, Norway; Clinic of Cardiology, St. Olavs Hospital, PO Box 3250, Torgarden, Trondheim NO-7006, Norway; Department of Circulation and Medical Imaging, Norwegian University of Science and Technology, PO Box 8905, Trondheim 7491, Norway; Department of Medicine, Drammen Hospital, Vestre Viken Hospital Trust, PO Box 800, Drammen 3004, Norway; Department of Behavioural Medicine, Faculty of Medicine, University of Oslo, PO Box 1078, Blindern, Oslo 0316, Norway; Faculty of Medicine, University of Oslo, PO Box 1078, Blindern, Oslo 0316, Norway; Department of Cardiology, Oslo University Hospital Ullevaal, PO Box 4950, Nydalen, Oslo 0424, Norway

**Keywords:** Cardiac disease, Cardiac symptoms, Quality of life

## Abstract

**Aims:**

Population-based data on cardiac-related symptoms in individuals without diagnosed cardiac disease remain limited, despite important implications for early detection and management. We aimed to assess the prevalence of self-reported symptoms and the association with quality of life (QoL) among adults aged ≥65 years with ≥1 additional stroke risk factor, but without diagnosed cardiac disease.

**Methods and results:**

This is a cross-sectional study of the NORwegian atrial fibrillation self-SCREENing (NORSCREEN) trial population at baseline. NORSCREEN is an ongoing, nationwide, randomized atrial fibrillation screening study in adults aged ≥65 years at increased risk of stroke (CHA_2_DS_2-_VA ≥2). All participants completed a baseline questionnaire capturing clinical information, symptoms, and QoL. Of the 50 549 participants enrolled from 2023 to 2025, 39 281 (78%) reported no diagnosed cardiac disease. Among those, 17 069 (43%) reported cardiac-related symptoms compared to 7551 (67%) of 11 268 individuals with known cardiac disease. The most common symptoms were fatigue, exertional dyspnoea, and tachycardia. Female sex [adjusted odds ratio 1.66, (95% CI 1.58–1.75)], physical inactivity [1.43 (1.32–1.55)], current smoking [1.24 (1.12–1.37)], age <75 years [1.14 (1.08–1.20)], living alone [1.13 (1.07–1.20)], and comorbidities including chronic obstructive pulmonary disease [6.51 (5.63–7.54)] and anxiety [3.99 (3.64–4.38)] were associated with cardiac-related symptoms. Symptomatic individuals reported significantly lower RAND-36 QoL scores across all domains compared to those without symptoms.

**Conclusion:**

In this cohort of individuals aged ≥65 years at increased risk of stroke, but without diagnosed cardiac disease, nearly half reported cardiac-related symptoms, which were associated with substantially reduced QoL. These findings suggest there might be unmet needs in identifying and managing cardiovascular disease.

**Registration:** Clinical trials: NCT05914883

## Graphical Abstract

**Graphical Abstract:**
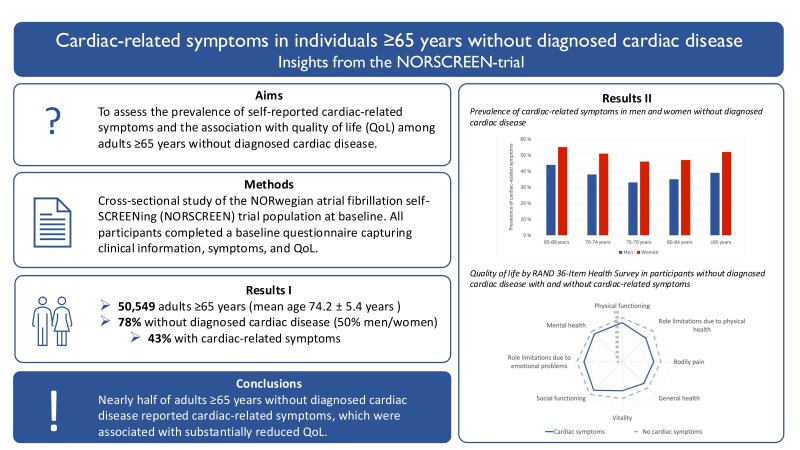

## Introduction

The prevalence of cardiac-related symptoms—such as tachycardia, palpitations, exertional dyspnoea, exertional chest pain, syncope, and episodes of fatigue—remains poorly characterized in adults without diagnosed cardiac disease. Since these symptoms can be non-specific, they also may originate from various non-cardiac aetiologies, such as pulmonary, metabolic, or psychological disorders. Assessment of symptoms is crucial even in individuals without known diseases, as symptom burden can impact quality of life (QoL) and may indicate underlying, unrecognized disease.

Physiological changes accompanying aging, such as reduced myocardial compliance, conduction system fibrosis, and altered autonomic regulation, may contribute to symptoms such as palpitations, dizziness, and exertional dyspnoea, even in the absence of overt cardiac disease^1,2^ These factors create diagnostic uncertainty, because symptoms may represent normal aging, undiagnosed disease, or both. Furthermore, unrecognized cardiac disease is common in older individuals, who may present with atypical or non-specific symptoms (e.g. fatigue instead of chest pain) contributing to delayed diagnosis and treatment.^3–5^

Robust, population-based estimates are needed to clarify how frequently such symptoms occur in individuals without diagnosed cardiac disease and to inform earlier detection of disease and efficient resource allocation. The present cross-sectional study aimed to (i) estimate the prevalence of self-reported cardiac-related symptoms and quality of life (QoL) and (ii) identify factors associated with cardiac-related symptoms, in a nationwide cohort of adults aged ≥65 years without diagnosed cardiac disease.

## Methods

### Study design

This exploratory, cross-sectional substudy was nested within the ongoing NORwegian atrial fibrillation self-SCREENing (NORSCREEN) trial—a nationwide, randomized controlled trial evaluating the impact of atrial fibrillation (AF) screening on stroke prevention in adults at increased risk of stroke.^6^ This analysis is based on baseline data collected at randomization. The study was reported in accordance with the Strengthening the Reporting of Observational Studies in Epidemiology (STROBE) guidelines.

### Study population

NORSCREEN is a siteless trial with digital recruitment from the general Norwegian population. The inclusion criteria were age 65 years or older and at least one additional risk factor for stroke according to the CHA_2_DS_2_-VA risk score [heart failure, hypertension, age ≥75 years (double), diabetes mellitus, previous stroke/transient ischaemic attack (double), and vascular disease].^7^ Exclusion criteria were self-reported history of AF, current use of anticoagulation therapy, implanted cardiac electronic devices, or no access to a smartphone. This analysis focused on participants without self-reported diagnosed cardiac disease.

### NORSCREEN study procedure

The participant selection process and participant inclusion in the NORSCREEN trial have been previously described in detail.^6^ In brief, adults aged ≥65 years were randomly identified from the Norwegian National Population Register and invited to participate in the NORSCREEN trial via secure digital messaging. After completing a digital eligibility assessment and providing digital informed consent, participants were randomized to either the screening or control group. The primary outcome of the NORSCREEN trial is time to first stroke.

At baseline, all participants completed a web-based questionnaire collecting demographic information as well as information about previous and chronic diseases, medication, cardiac-related symptoms, and QoL (*Table 1*). This information formed the basis for the present cross-sectional substudy. All baseline information was self-reported.

**Table 1:** Self-reported baseline information

Category	Variables
Demographic information	Sex, age, weight, height, education, employment status, cohabiting status, smoking, alcohol consumption, physical activity level
Previous conditions	Diabetes mellitus, hypertension, stroke, peripheral artery disease, arrhythmias, heart failure, coronary artery disease (including myocardial infarction, percutaneous coronary intervention, and/or coronary artery bypass grafting), hypo- or hyperthyroidism, chronic obstructive pulmonary disease (COPD), obstructive sleep apnoea, anxiety disorder
Medication use	Current use of antiplatelet agents, lipid lowering therapy, beta-blockers, angiotensin-converting enzyme (ACE) inhibitors, angiotensin II receptor antagonists
Cardiac-related symptoms	Tachycardia, palpitations, exertional dyspnoea, exertional chest pain, syncope, fatigue
Quality of life (RAND-36)	Physical functioning, role limitations due to physical health, bodily pain, general health perceptions, vitality, social functioning, role limitations due to emotional problems, mental health

### Definitions and questionnaires

All participants who reported any prior diagnosis of arrhythmias, heart failure, or coronary artery disease (myocardial infarction, percutaneous coronary intervention, and/or coronary artery bypass grafting) were classified as having cardiac disease. Individuals who did not report any of these conditions were defined as participants without diagnosed cardiac disease.

Cardiac-related symptoms refer to self-reported sensations that may suggest a cardiac origin, experienced at any time before inclusion in the study. These include tachycardia, palpitations, exertional dyspnoea, exertional chest pain, syncope, and episodes of fatigue. Symptoms were assessed via self-reporting using a standardized questionnaire developed for this study (see Supplementary material online, *File S1*).

QoL was assessed using the RAND 36-Item Health Survey (RAND-36), a validated generic instrument measuring health-related quality of life across eight domains: physical functioning, role limitations due to physical health, bodily pain, general health perceptions, vitality, social functioning, role limitations due to emotional problems, and mental health.^8,9^ Each domain score ranges from 0 to 100, with higher scores indicating better self-reported health status. A composite profile was generated by calculating the mean score within each domain, following standard RAND-36 scoring procedures.^8,9^ The questionnaire reflects participants’ perceived health status during the 4 weeks prior to survey completion.

### Outcomes and follow-up

The primary outcome of this substudy was the prevalence of one or more cardiac-related symptoms at study inclusion among participants without prior diagnosed cardiac disease. Secondary outcomes included the prevalence of each individual symptom and associations between symptom presence and baseline characteristics. Additionally, QoL was evaluated in individuals both with and without cardiac-related symptoms. This study did not involve any follow-up.

### Patient and public involvement

The Steering Committee of the NORSCREEN trial includes a user representative who was involved in the preparation of the study protocol.

### Statistics

Descriptive statistics were used to summarize participant characteristics and symptom prevalence. Continuous variables were expressed as mean ± standard deviation (SD) or median (IQR), as appropriate. Categorical variables were summarized as frequencies and percentages. The proportions were calculated based on non-missing data. A multivariable logistic regression model was used to identify factors independently associated with self-reported cardiac-related symptoms. Covariates included sex, age, obesity, education, cohabiting, current smoking, alcohol use, daily physical activity, and comorbidity. Results are reported as adjusted odds ratios (aOR) with 95% confidence intervals (CI). Subgroup analyses were performed across age, sex, and specific comorbid conditions. A *P*-value <0.05 was considered statistically significant. All data were analysed with STATA (Version 18).

### Ethics

The study was conducted in accordance with the Declaration of Helsinki. The study protocol is consistent with Good Clinical Practice (GCP) and applicable regulatory requirements. All patients provided signed informed consent prior to study participation. The study protocol, including the patient information and informed consent form, was approved by the Norwegian Committee for Medical and Health Research Ethics (477781) and by the Data Protection Officer at Oslo University Hospital.

## Results

**Figure 1:**
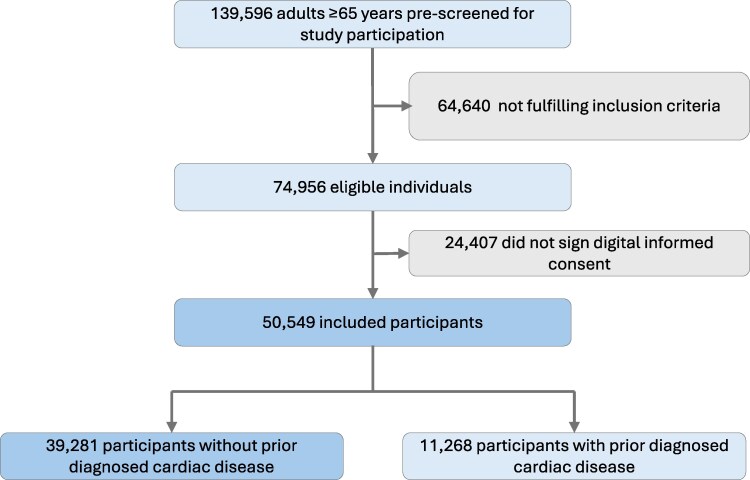
Study population creation.

A total of 50 549 adults aged ≥65 years were included in the NORSCREEN trial from September 2023 to June 2025 (*Figure 1*).

No prior diagnosed cardiac disease (e.g. arrhythmia, heart failure, or coronary artery disease) was reported at baseline in 39 281 (77.7%) participants. Baseline clinical characteristics of participants with and without diagnosed cardiac disease are presented in *Table 2*. There were no significant differences in age between the groups.

**Table 2:** Clinical characteristics in adults aged ≥65 years with and without diagnosed cardiac disease (*n* = 50 549)

Characteristics	Diagnosed cardiac disease	No diagnosed cardiac disease
	*n* = 11 268	*n* = 39 281
Women, *n*	3777/11 268 (33.5%)	19 668/39 281 (50.1%)
Mean age [years (SD)]	74.2 ± 5.6	74.2 ± 5.4
Median age [years (IQR)]	74 (69, 78)	75 (69, 78)
Body mass index [mean, kg/m^2^ (SD)]	26.6 ± 3.9	26.5 ± 4.2
Obesity (body mass index ≥30 kg/m^2^)	1989/11 268 (17.7%)	7081/39 277 (18.0%)
Higher education, *n*	5755/11 262 (51.1%)	20 609/39 264 (52.5%)
Employed, *n*	1379/11 264 (12.2%)	4559/39 280 (11.6%)
Living alone, *n*	2745/11 268 (24.4%)	10 481/39 273 (26.7%)
Current smoker, *n*	809/11 268 (7.2%)	2531/39 277 (6.4%)
Weekly alcohol use, *n*	5228/11 268 (46.4%)	19 264/39 277 (49.1%)
Daily physical activity <30 min, *n*	1391/11 268 (12.3%)	4106/39 277 (10.5%)
Previous diseases		
Diabetes mellitus, *n*	1715/11 098 (15.5%)	4974/38 825 (12.8%)
Hypertension, *n*	7034/10 941 (64.3%)	26 049/38 372 (67.9%)
Stroke, *n*	701/11 075 (6.3%)	2351/38 658 (6.1%)
Peripheral artery disease, *n*	514/11 204 (4.6%)	657/39 128 (1.7%)
Hypothyroidism, *n*	1039/11 023 (9.4%)	3818/38 885 (9.8%)
Hyperthyroidism, *n*	185/11 037 (1.7%)	448/38 922 (1.2%)
Chronic obstructive pulmonary disease, *n*	702/10 988 (6.4%)	1659/38 490 (4.3%)
Obstructive sleep apnoea disorder, *n*	1553/10 104 (15.4%)	3758/35 627 (10.6%)
Anxiety, *n*	1394/10 862 (12.8%)	3379/37 824 (8.9%)
Medication		
Platelet inhibitor, *n*	7470/11 203 (66.7%)	7456/39 019 (19.1%)
Lipid lowering therapy, *n*	8615/11 225 (76.8%)	17 690/39 119 (45.2%)
Beta-blockers, *n*	3862/11 085 (34.8%)	2698/38 859 (6.9%)
Angiotensin-converting enzyme inhibitor, *n*	1509/11 044 (13.7%)	2240/38 882 (5.8%)
Angiotensin II receptor antagonist, *n*	2926/11 015 (26.6%)	10 151/38 756 (26.2%)

### Prevalence of cardiac-related symptoms

A total of 17 069 (43.5%) participants without diagnosed cardiac disease reported cardiac-related symptoms, compared to 7551 (67.0%) participants with known cardiac disease (*Table 3*). Fatigue was the most common symptom in both groups. Symptomatic participants with no history of cardiac disease reported an average of 1.8 (SD 1.0) distinct symptoms, while those with cardiac disease reported an average of 2.2 (SD 1.2) symptoms.

**Table 3:** Cardiac-related symptoms in adults aged ≥65 years with and without diagnosed cardiac disease

	Diagnosed cardiac disease	No diagnosed cardiac disease
	*n* = 11 268	*n* = 39 281
Any cardiac symptom, *n*	7551/11 268 (67.0%)	17 069/39 274 (43.5%)
Fatigue, *n*	4001/10 461 (38.3%)	9426/36 455 (25.9%)
Exertional dyspnoea, *n*	3350/10 654 (31.4%)	6971/36 918 (18.9%)
Tachycardia, *n*	3035/10 568 (28.7%)	5702/36 586 (15.6%)
Palpitations, *n*	3647/10 426 (35.0%)	5053/36 042 (14.0%)
Syncope, *n*	960/11 109 (8.6%)	2578/38 782 (6.7%)
Exertional chest pain, *n*	1378/10 916 (12.6%)	1065/38 504 (2.8%)

The presence of specific cardiac-related symptoms in participants without diagnosed cardiac disease across other comorbidities is listed in *Table 4*. Participants without any comorbidity were less likely to report cardiac-related symptoms (1775 out of 6260 individuals; 28.4%) compared to those with at least one pre-existing non-cardiac condition (14 542 out of 31 346; 46.4%). Fatigue was the most prevalent symptom in previously healthy people.

**Table 4:** Cardiac-related symptoms in adults aged ≥65 years without diagnosed cardiac disease across other comorbidities

	No previous disease	Diabetes	Hypertension	Stroke	Hypothyroidism	Chronic obstructive pulmonary disease	Obstructive sleep apnoea disorder	Anxiety
	*n* = 6260	*n* = 4974	*n* = 26 049	*n* = 2351	*n* = 3.818	*n* = 1659	*n* = 3758	*n* = 3379
Any cardiac symptom, *n*	1775/6260 (28.4%)	2311/4974 (46.5%)	11 861/26 047 (45.5%)	1193/2351 (50.7%)	2104/3818 (55.1%)	1366/1659 (82.3%)	2103/3758 (56.0%)	2551/3379 (75.5%)
Fatigue, *n*	801/5955 (13.5%)	1524/4602 (32.9%)	6525/24 179 (27.0%)	762/2187 (34.8%)	1353/3539 (38.2%)	803/1541 (52.1%)	1478/3499 (42.2%)	1833/3144 (58.3%)
Exertional dyspnoea, *n*	495/6030 (8.2%)	1005/4664 (21.6%)	5020/24 427 (20.6%)	529/2233 (23.7%)	848/3556 (23.9%)	1174/1567 (74.9%)	1072/3502 (30.6%)	1066/3129 (34.1%)
Tachycardia, *n*	474/6017 (7.9%)	674/4639 (14.5%)	4196/24 166 (17.4%)	368/2192 (16.8%)	726/3502 (20.7%)	374/1536 (24.4%)	645/3496 (18.5%)	1105/3008 (36.7%)
Palpitations, *n*	496/5946 (8.3%)	581/4610 (12.6%)	3622/23 826 (15.2%)	299/2172 (13.8%)	643/3419 (18.8%)	299/1505 (19.9%)	569/3440 (16.5%)	900/2923 (30.8%)
Syncope, *n*	393/6198 (6.3%)	291/4914 (5.9%)	1623/25 738 (6.3%)	193/2307 (8.4%)	297/3763 (7.9%)	124/1635 (7.6%)	236/3707 (6.4%)	391/3306 (11.8%)
Exertional chest pain, *n*	96/6208 (1.6%)	161/4873 (3.3%)	707/25 529 (2.8%)	90/2307 (3.9%)	149/3730 (4.0%)	124/1593 (7.8%)	195/3658 (5.3%)	209/3239 (6.5%)

**Figure 2:**
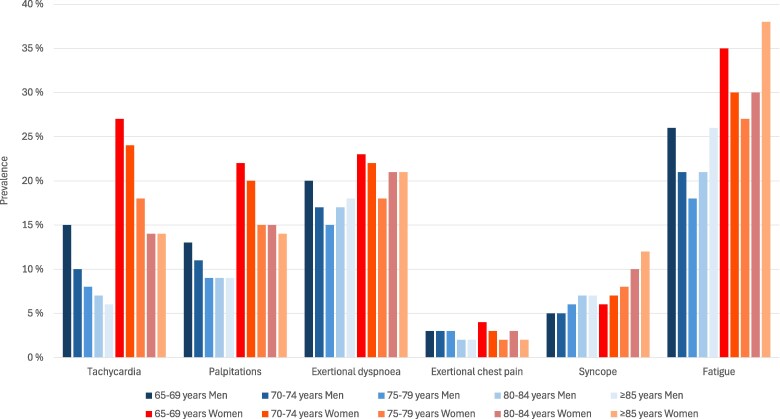
Age- and sex-specific prevalence of various cardiac-related symptoms among adults aged ≥65 years without diagnosed cardiac disease (*n* = 39 281).

*Figure 2* displays the age- and sex-specific prevalences of various cardiac-related symptoms among adults aged ≥65 years without diagnosed cardiac disease. Clinical characteristics in symptomatic men and women without diagnosed cardiac disease are presented in Supplementary material online, *Table S1*. Across all age strata, women consistently reported a higher prevalence of symptoms compared to men. Fatigue was the most frequently reported symptom, with prevalence rates increasing with age and peaking at 38% among women aged ≥85 years. Tachycardia and palpitations were also more commonly reported by women, particularly in the younger individuals (65–69 years), with a prevalence of 27%, compared to 15% in men of the same age group. The prevalence of tachycardia and palpitations declined progressively with advancing age in both sexes. Exertional dyspnoea showed relatively stable prevalence across age groups, with women reporting slightly higher rates (18–23%) than men (15–20%). Exertional chest pain was infrequently reported across all groups, with prevalence remaining below 4% in both sexes. Syncope demonstrated a modest age-related increase, particularly among women, rising from 6% in the youngest group to 12% in those aged ≥85 years.

### Factors associated with cardiac-related symptoms in participants without diagnosed cardiac disease

Female sex, physical inactivity (<30 min physical activity daily), current smoking, obesity, age <75 years, living alone, and comorbidity, especially chronic obstructive pulmonary disease (COPD) and anxiety, were significantly associated with the presence of cardiac-related symptoms among participants without diagnosed cardiac disease (*Table 5*). Similar patterns were observed for the individual symptom tachycardia, palpitations, exertional dyspnoea, exertional chest pain, syncope, and fatigue (see Supplementary material online, *Table S2*).

**Table 5:** Factors associated with cardiac-related symptoms among adults aged ≥65 years without diagnosed cardiac disease

	Odds ratio (95% confidence interval)	Adjusted odds ratio (95% confidence interval)^a^
Female sex	1.69 (1.62–1.76)	1.66 (1.58–1.75)
Daily physical activity <30 min	1.71 (1.60–1.82)	1.43 (1.32–1.55)
Current smoking	1.61 (1.48–1.74)	1.24 (1.12–1.37)
Obesity (body mass index ≥30 kg/m^2^)	1.51 (1.43–1.59)	1.18 (1.11–1.26)
Age <75 years	1.31 (1.25–1.36)	1.14 (1.08–1.20)
Living alone	1.39 (1.33–1.45)	1.13 (1.07–1.20)
Higher education	0.85 (0.81–0.88)	1.01 (0.97–1.06)
Employment	0.94 (0.89–1.00)	0.97 (0.90–1.04)
Weekly alcohol use	0.81 (0.77–0.84)	0.93 (0.89–0.98)
Comorbidity		
Chronic obstructive pulmonary disease	6.69 (5.89–7.60)	6.51 (5.63–7.54)
Anxiety	4.68 (4.31–5.07)	3.99 (3.64–4.38)
Hyperthyroidism	2.41 (1.98–2.92)	1.77 (1.41–2.21)
Obstructive sleep apnoea disorder	1.88 (1.75–2.01)	1.64 (1.52–1.78)
Stroke	1.38 (1.27–1.50)	1.46 (1.33–1.62)
Peripheral artery disease	1.52 (1.30–1.77)	1.41 (1.17–1.70)
Hypothyroidism	1.70 (1.59–1.82)	1.29 (1.19–1.40)
Hypertension	1.34 (1.29–1.40)	1.24 (1.18–1.31)
Diabetes mellitus	1.16 (1.09–1.23)	1.11 (1.03–1.19)

^a^Adjusted for all listed factors.

Even among participants without any reported previous diseases (*n* = 6260), age <75 years (aOR 1.88, 95% CI 1.53–2.32), female sex (aOR 1.63, 95% CI 1.45–1.84), obesity (aOR 1.39 (95% CI 1.10–1.75), physical inactivity (aOR 1.35, 95% CI 1.07–1.70), and living alone (aOR 1.15, 95% CI 1.02–1.31) were independently associated with cardiac-related symptoms.

### Quality of life in participants without diagnosed cardiac disease

**Figure 3:**
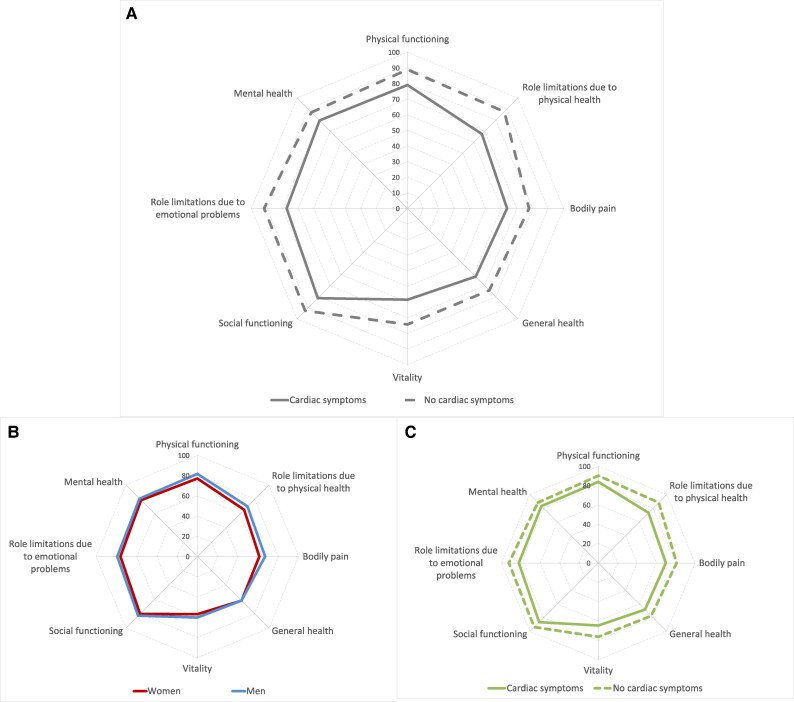
(*A*) Quality of life by RAND 36-item health survey in participants without diagnosed cardiac disease with and without cardiac-related symptoms (*B*) Quality of life by RAND 36-Item Health Survey in symptomatic men and women without diagnosed cardiac disease (*C*) Quality of life by RAND 36-Item Health Survey in participants without any diagnosed disease with and without cardiac-related symptoms.

Symptomatic adults aged ≥65 years without diagnosed cardiac disease had significantly lower scores across all RAND-36 domains compared to those without symptoms (*Figure 3A*). The largest differences were observed in the domains ‘role limitations due to physical health’ (67.4 vs. 87.6) and ‘vitality’ (58.4 vs. 74.3). Symptomatic women without diagnosed cardiac disease had slightly lower RAND-36 domain scores than their male counterparts across most dimensions (*Figure 3B*). Even those *individuals* without any diagnosed diseases at all, but reporting cardiac-related symptoms, had significantly lower scores across all RAND-36 domains compared to those without symptoms (*Figure 3C*).

## Discussion

This large cross-sectional study of the NORSCREEN trial population at baseline provides additional insights into the burden of cardiac-related symptoms among adults aged ≥65 years at elevated stroke risk but without prior cardiac diagnosis. Nearly half of these individuals reported one or more cardiac-related symptoms, with fatigue being the most common, followed by exertional dyspnoea and tachycardia. The symptom burden was strongly associated with female sex, age <75 years, obesity, comorbidities (notably COPD and anxiety), physical inactivity, and social determinants such as living alone. Furthermore, cardiac-related symptoms were consistently linked to poorer QoL across all RAND-36 domains. These findings challenge the assumption that absence of a cardiac diagnosis equates to absence of cardiovascular symptoms, suggesting substantial unmet needs in identifying and managing cardiovascular diseases.

In participants without previously diagnosed cardiac disease, the prevalence of cardiac-related symptoms was notably high at 43.5%, compared with 67.0% among those with known cardiac disease. While previous studies have reported symptom prevalence in general cohorts, few studies have systematically examined symptom patterns, associated determinants, and QoL implications in individuals without diagnosed cardiac disease at this scale. Our observation that fatigue constitutes a predominant self-reported cardiac-related symptom in individuals without diagnosed cardiac disease aligns with prior reports in broader populations. A recent meta-analysis estimated an overall prevalence of fatigue at 42.6% in older adults, and the US Health and Retirement Study data reported 31.2%, with higher rates in women.^10,11^ Dyspnoea of at least moderate severity affects roughly one-third of adults aged 65 years and older in the general population.^12,13^ Fatigue and dyspnoea are not specific to cardiac disease and frequently occur in other diseases, including pulmonary, kidney, haematological, musculoskeletal, and psychological disorders. Hence, we cannot exclude that the high prevalence of fatigue and dyspnoea might be due to undiagnosed non-cardiac diseases.

In the study population without diagnosed cardiac disease, other possible cardiac-related symptoms were also prevalent; tachycardia was reported by 15.6% of participants, palpitations by 14.0%, syncope by 6.7%, and exertional chest pain by 2.8%. These findings are consistent with the known prevalence of such symptoms in the general ageing population^14–16^ Chest pain has been more extensively characterized in clinical than in community settings. In the US Cardiovascular Health Study of community-dwelling adults aged 65 years or older, the prevalence of angina ranged from ∼15–17% in men and 8–13% in women, with rates rising with advancing age.^17^

The relationship between cardiac-related symptoms, demographic factors, and comorbidity is complex and multifaceted. Women reported more cardiac-related symptoms than men, which may be due to differences in symptom perception, health-seeking behaviour, and a higher prevalence of comorbidities.^18,19^ Individuals with multiple comorbid conditions, particularly COPD and anxiety, were more likely to report cardiac-related symptoms even in the absence of diagnosed cardiac disease. For example, the subgroup with known COPD had a notably higher prevalence of exertional dyspnoea, a symptom that most likely originates from the COPD itself rather than cardiac causes alone. A similar trend was observed among those with hypertension and diabetes—both well-established risk factors for coronary heart disease and heart failure with preserved ejection fraction (HFpEF). Comorbidities may complicate the clinical picture because overlapping symptoms could be attributed to non-cardiac conditions, which might result in reduced recognition of possible cardiac disease.^19^

Several studies have reported substantial rates of unrecognized structural heart disease in older individuals.^4,5^ Moreover, older individuals frequently present with atypical cardiac symptoms, such as fatigue, rather than chest pain, which complicates early detection and may delay appropriate care.^3,20^ The presence of multiple comorbidities and polypharmacy in older populations can obscure the clinical picture, as symptoms may overlap or be erroneously ascribed to non-cardiac origins.^20^ It remains uncertain whether the absence of self-reported cardiac disease truly reflects the absence of underlying heart conditions, and we do not have follow-up data to assess the eventual development of cardiac disease in these individuals.

Nonetheless, clinicians should be especially vigilant when evaluating unexplained symptoms in older adults.

The persistent association between self-reported cardiac-related symptoms and diminished health-related QoL, as measured across all domains of the RAND-36 instrument, suggests that symptom burden may serve as a proxy for broader functional decline and psychosocial distress. This highlights the importance of early identification and comprehensive management of symptomatic individuals, even when traditional diagnostic criteria for cardiac disease are not met.

### Limitations

Although the study benefits from a large sample size, several important limitations should be acknowledged. The cross-sectional design precludes any causal inference between the reported cardiac-related symptoms and the associated demographic and clinical factors. Moreover, the cross-sectional design does not capture changes over time, making it impossible to assess the progression of symptoms or the development of cardiac disorders in individual participants. The study lacks information regarding the timing of cardiac-related symptoms and the temporal relationship between cardiac-related symptoms and coexisting conditions, as well as details regarding symptom duration and severity. As the NORSCREEN trial is a population-based AF screening study, the inclusion criteria prioritize individuals with comorbidities (CHA₂DS₂-VA score ≥2), while those with known AF, implanted pacemakers/ICDs, and use of anticoagulation therapy were excluded. Participation required access to and familiarity with digital tools, which may have introduced selection bias by excluding individuals with limited technological proficiency. Frail or institutionalized individuals who may have a higher symptom burden are likely underrepresented. Additionally, there is also a possibility of healthy participant bias, as individuals who consent to screening may be more health-conscious or have different perceptions of symptoms than those who do not participate. These factors affect the generalizability of the findings to the broader population. All data were self-reported, which introduces the possibility of recall bias and subjective interpretation. Some participants may underreport or overreport diseases and symptoms due to individual variation in health perception, literacy, or expectations. In addition, the definition of diseases and cardiac-related symptoms was based on general descriptions and not confirmed by clinical or diagnostic evaluations. Furthermore, no validated symptom questionnaire was available in Norwegian. Individuals with hypertension and diabetes were not classified as having established cardiac disease, and valvular heart disease was not documented. Potential cultural and linguistic variations in symptom interpretation and reporting were not systematically accounted for, which may influence the observed prevalence rates. Unfortunately, data regarding key comorbidities, including chronic kidney disease, anaemia, and malignancy, were not available. Furthermore, echocardiographic data were not available, precluding assessment of undiagnosed structural heart disease [e.g. heart failure with preserved ejection fraction (HFpEF)]. Consequently, the presence of residual confounding cannot be ruled out. Known sex differences in RAND-36 scores may influence our findings, and this should be considered when interpreting the results.^21^ Lastly, the findings may reflect the specific healthcare, social, and demographic context of Norway, and may not be directly generalizable to countries with different population structures or healthcare systems.

## Conclusions

This cross-sectional study demonstrates a high prevalence of cardiac-related symptoms among adults aged ≥65 years at elevated stroke risk despite no prior cardiac diagnosis and highlights their significant association with reduced QoL. Recognizing symptom prevalence not only highlights unmet clinical needs but also aids clinicians in evaluating these complaints, supporting comprehensive assessment and earlier detection of possible undiagnosed cardiac conditions. Future studies are needed to evaluate the prevalence and implications of unrecognized heart disease in individuals with cardiac-related symptoms but without prior diagnosed cardiac disease.

## Supplementary Material

oeag032_Supplementary_Data

## Data Availability

The data underlying this article will be shared on reasonable request to the corresponding author.
