# Supplementary material for: Cardiac-related symptoms in individuals aged ≥65 years without diagnosed cardiac disease: insights from the NORSCREEN trial
Source: Eur Heart J Open. 2026 Feb 20;6(2):oeag032. doi: 10.1093/ehjopen/oeag032 (PMC12987703; doi:10.1093/ehjopen/oeag032)
Supplement: oeag032_Supplementary_Data [file oeag032_supplementary_data.zip › Supplementary tables.docx]

| **Supplementary table 1. Clinical characteristics in symptomatic men and women ≥65 years without diagnosed cardiac disease (n=17,069)** | | | | | | | | | | | | | | |
| --- | --- | --- | --- | --- | --- | --- | --- | --- | --- | --- | --- | --- | --- | --- |
| **Characteristics** | | | | | **Men** | | | | | **Women** | | | | |
|  | | | | | n=7,268 | | | | | n=9,801 | | | | |
|  | | | | |  | | | | |  | | | | |
| Mean age (years (SD)) | | | | | 73.7 ± 5.6 | | | | | 73.9 ± 5.4 | | | | |
| Median age (years (IQR) | | | | | 74 (69, 78) | | | | | 74 (69, 78) | | | | |
| Body mass index (mean, kg/m^2^ (SD)) | | | | | 27.2 ± 4.1 | | | | | 26.6 ± 4.7 | | | | |
| Obesity (body mass index ≥30 kg/m^2^) | | | | | 1,641/7,268 (22.6%) | | | | | 2,027/9,801 (20.7%) | | | | |
| Higher education, n | | | | | 3,743/7,267 (51.5%) | | | | | 4,808/9,796 (49.1%) | | | | |
| Employed, n | | | | | 1,165/7,268 (16.0%) | | | | | 759/9,801 (7.7%) | | | | |
| Living alone, n | | | | | 1,424/7,266 (19.6%) | | | | | 3,754/9,798 (38.3%) | | | | |
| Current smoker, n | | | | | 641/7,268 (8.8 %) | | | | | 739/9,801 (7.5 %) | | | | |
| Weekly alcohol use, n | | | | | 3,865/7,268 (53.2%) | | | | | 3,984/9,801 (40.7%) | | | | |
| Daily physical activity <30 min, n | | | | | 1,165/7,268 (16.0%) | | | | | 1,108/9,801 (11.3%) | | | | |
| Previous diseases | | | | |  | | | | |  | | | | |
| Diabetes mellitus, n | | | | | 1,243/7,157 (17.4%) | | | | | 1,068/9,663 (11.1%) | | | | |
| Hypertension, n | | | | | 5,069/7.050 (71.9%) | | | | | 6,792/9,540 (71.2%) | | | | |
| Stroke, n | | | | | 588/7,080 (8.3%) | | | | | 605/9,604 (6.3%) | | | | |
| Peripheral artery disease, n | | | | | 178/7,239 (2.5%) | | | | | 174/9,757 (1.8%) | | | | |
| Hypothyroidism, n | | | | | 354/7,124 (5.0%) | | | | | 1,750/9,691 (18.1%) | | | | |
| Hyperthyroidism, n | | | | | 56/7,140 (0.8%) | | | | | 233/9,706 (2.4%) | | | | |
| Chronic obstructive pulmonary disease, n | | | | | 704/7,009 (10.0%) | | | | | 662/9,481 (6.7%) | | | | |
| Obstructive sleep apnoea disorder, n | | | | | 1,210/6,319 (19.2%) | | | | | 893/8,650 (10.3%) | | | | |
| Anxiety, n | | | | | 936//6,954 (13.5%) | | | | | 1,615/9,275 (17.4%) | | | | |
| Medication | | | | |  | | | | |  | | | | |
| Platelet inhibitor, n | | | | | 1,868/71,74 (26.0%) | | | | | 1,840/9,742 (18.9%) | | | | |
| Lipid lowering therapy, n | | | | | 3,680/7,222 (51.0%) | | | | | 4,331/9,762 (44.4%) | | | | |
| Beta-blockers, n | | | | | 568/7,147 (8.0%) | | | | | 1,011/9,685 (10.4%) | | | | |
| Angiotensin-converting enzyme inhibitor, n | | | | | 519/7,145 (7.3%) | | | | | 523/9,694 (5.4%) | | | | |
| Angiotensin II receptor antagonist, n | | | | | 1,926/7,116 (27.1%) | | | | | 2,753/9,686 (28.4%) | | | | |
| **Supplementary table 1. Adjusted* odds ratio (OR) with 95% confidence interval for individual cardiac-related symptom among adults ≥65 years without diagnosed cardiac disease** | | | | | | | | | | | | | | |
|  | **Tachycardia** | | **Palpitations** | | | **Exertional dyspnoea** | | **Exertional chest pain** | | | **Syncope** | | **Fatigue** | |
| Female sex | 2.43 | (2.25-2.61) | 1.84 | (1.70-1.98) | | 1.31 | (1.22-1.41) | 0.86 | (0.73-1.00) | | 1.37 | (1.24-1.52) | 1.48 | (1.39-1.58) |
| Daily physical activity <30 min | 0.94 | (0.84-1.05) | 0.91 | (0.81-1.03) | | 1.73 | (1.57-1.90) | 1.19 | (0.96-1.48) | | 1.17 | (1.00 -1.35) | 1.59 | (1.46-1.74) |
| Current smoking | 1.12 | (0.98-1.28) | 0.97 | (0.84-1.12) | | 1.30 | (1.15-1.47) | 0.94 | (0.71-1.25) | | 1.02 | (0.84-1.23) | 1.31 | (1.17-1.47) |
| Obesity (body mass index ≥30 kg/m^2^) | 1.01 | (0.94-1.08) | 0.86 | (0.78-0.95) | | 2.01 | (1.86-2.18) | 1.22 | (1.01-1.46) | | 0.70 | (0.61-0.81) | 1.28 | (1.19-1.38) |
| Age <75 years | 1.50 | (1.39-1.62) | 1.38 | (1.28-1.50) | | 1.02 | (0.96-1.10) | 1.25 | (1.06-1.47) | | 0.82 | (0.74-0.91) | 1.07 | (1.00-1.14) |
| Living alone | 1.01 | (0.93-1.06) | 1.00 | (0.92-1.08) | | 1.24 | (1.15-1.33) | 1.00 | (0.84-1.18) | | 1.05 | (0.95-1.17) | 1.26 | (1.18-1.34) |
| Higher education | 1.01 | (0.92-1.11) | 1.16 | (1.08-1.24) | | 2.01 | (1.86-2.18) | 1.22 | (1.01-1.46) | | 0.70 | (0.61-0.81) | 1.28 | (1.19-1.38) |
| Employment | 1.05 | (0.95-1.17) | 0.92 | (0.82-1.03) | | 1.04 | (0.94-1.16) | 0.87 | (0.69-1.10) | | 1.01 | (0.86-1.18) | 0.95 | (0.86-1.04) |
| Weekly alcohol use | 1.04 | (0.97-1.12) | 0.99 | (0.92-1.06) | | 0.95 | (0.89-1.02) | 0.82 | (0.70-0.95) | | 1.04 | (0.94-1.14) | 0.76 | (0.72-0.81) |
| Comorbidity |  |  |  |  | |  |  |  |  | |  |  |  |  |
| Chronic obstructive pulmonary disease | 1.69 | (1.46-1.97) | 1.47 | (1.25-1.73) | | 15.11 | (13.19-17.31) | 2.37 | (1.84-3.04) | | 1.10 | (0.88-1.37) | 2.89 | (2.55-3.27) |
| Anxiety | 3.43 | (3.12-3.77) | 2.91 | (2.64-3.22) | | 2.05 | (1.85-2.26) | 2.38 | (1.97-2.88) | | 1.98 | (1.74-2.27) | 4.30 | (3.93-4.69) |
| Hyperthyroidism | 1.62 | (1.26-2.10) | 1.39 | (1.04-1.83) | | 1.72 | (1.33-2.22) | 1.42 | (0.82-2.46) | | 1.65 | (1.18 -2.31) | 1.79 | (1.42-2.26) |
| Obstructive sleep apnoea disorder | 1.31 | (1.18-1.46) | 1.34 | (1.20-1.50) | | 1.67 | (1.51-1.84) | 1.80 | (1.48-2.18) | | 1.03 | (0.88-1.20) | 2.17 | (1.99-2.37) |
| Stroke | 1.18 | (1.03-1.35) | 1.04 | (0.91-1.20) | | 1.43 | (1.26-1.62) | 1.40 | (1.08-1.81) | | 1.38 | (1.16-1.64) | 1.68 | (1.51-1.88) |
| Peripheral artery disease | 1.13 | (0.87-1.46) | 1.02 | (0.77-1.35) | | 1.53 | (1.22-1.93) | 1.58 | (1.01-2.49) | | 1.21 | (0.86-1.70) | 1.44 | (1.17-1.77) |
| Hypothyroidism | 1.05 | (0.94-1.17) | 1.15 | (1.03-1.84) | | 1.17 | (1.05-1.30) | 1.56 | (1.26-1.93) | | 1.11 | (0.96-1.28) | 1.46 | (1.33-1.60) |
| Hypertension | 1.33 | (1.23-1.45) | 1.26 | (1.16-1.38) | | 1.29 | (1.19-1.39) | 0.85 | (0.72-1.00) | | 1.03 | (0.93-1.15) | 1.13 | (1.06-1.21) |
| Diabetes mellitus | 0.92 | (0.83-1.03) | 0.91 | (0.82-1.01) | | 0.99 | (0.90-1.09) | 1.04 | (0.84 -1.27) | | 1.00 | (0.86-1.149) | 1.37 | (1.27-1.49) |

*Adjusted for all listed factors
