# Supplementary material for: Cardiac-related symptoms in individuals aged ≥65 years without diagnosed cardiac disease: insights from the NORSCREEN trial
Source: Eur Heart J Open. 2026 Feb 20;6(2):oeag032. doi: 10.1093/ehjopen/oeag032 (PMC12987703; doi:10.1093/ehjopen/oeag032)
Supplement: oeag032_Supplementary_Data [file oeag032_supplementary_data.zip › Supplementary files.docx]

Supplementary file 1

NORSCREEN QUESTIONNAIRE

# Introduction

Thank you for your interest in participating in the NORSCREEN study!

We would like to know more about you, your risk factors for atrial fibrillation, previous heart and vascular diseases, and your medications.

All participants will receive similar questionnaires every 6 months during the study period.

Half of the participants will also be randomly selected to perform an ECG test at home.

Your answers are very important for the research project – thank you for taking a few minutes to complete this questionnaire.

After completing this form, click Send to proceed to the consent form for participation in the study. The consent must be signed with BankID.

# Demographics

- Birth number (11 digits)
- Name (first and last)
- Mobile number
- Email address
- Gender: Female, Male, Other, Prefer not to say
- Age (years)
- Height (cm)
- Weight (kg)

# Education

- Primary school
- Secondary school
- University/college <4 years
- University/college 4 years
- Other
- Don’t know

# Employment Status

- Retired
- Employed – full-time
- Employed – part-time
- Disabled
- On sick leave
- Other
- Don’t know

# Marital Status

- Married/cohabiting
- Widow/widower
- Single
- Other
- Don’t know

# Income

- <200,000 NOK
- 200,000 – 400,000 NOK
- 400,000 – 800,000 NOK
- >800,000 NOK
- Don’t know

# Smoking and Alcohol

- Do you smoke? Never smoked, Quit, Occasionally, Daily
- Alcohol in last 12 months? Yes/No
- Frequency: Daily, Weekly, Monthly, Less than monthly

# Diseases

# Have you had or do you have any of the following illnesses/conditions? Answer YES only for illnesses/conditions that have been diagnosed by a doctor/hospital. Yes/No/Don’t know

- Other rhythm disorders (not atrial fibrillation)
- Myocardial infarction
- Diabetes
- High blood pressure
- Stroke
- Coronary heart artery treatment (PCI or surgery)
- Leg artery treatment
- Heart failure
- Thyroid disorders
- COPD
- Sleep apnea

# Symptoms

# Have you had or do you have any of the following symptoms? Yes/No/Don’t know

- Rapid heartbeat
- Irregular heartbeat
- Syncope
- Shortness of breath
- Chest pain
- Fatigue
- Anxiety

# Medications

Do you use any of the following medications daily? Yes/No/Don’t know

- Acetylsalicylic acid
- Statins
- Beta-blockers
- Sotalol
- Flecainide
- Dronedarone
- Amiodarone
- ACE inhibitors
- AII blockers

# Physical Activity

- 30 min daily activity? Yes/No
- Exercise frequency: Never, <1/week, 1/week, 2–3/week, Almost daily
- Intensity: Easy, Moderate, Hard
- Duration: <15 min, 15–29 min, 30–60 min, >60 min

# Rand-36 Health

- In general, would you say your health is: Excellent, Very good, Good, Fair, Poor
- **Compared to one year ago**, how would you rate your health in general **now**? Much better, Somewhat better, Same, Somewhat worse, Much worse
- The following items are about activities you might do during a typical day. Does **your health now limit you** in these activities? If so, how much? Yes, limited a lot/Yes, limited a little/No, not limited at all
- **Vigorous activities**, such as running, lifting heavy objects, participating in strenuous sports
- **Moderate activities**, such as moving a table, pushing a vacuum cleaner, bowling, or playing golf
- Lifting or carrying groceries
- Climbing **several** flights of stairs
- Climbing **one** flight of stairs
- Bending, kneeling, or stooping
- Walking **more than 2 kilometers**
- Walking **several blocks**
- Walking **one block**
- Bathing or dressing yourself
- During the **past 4 weeks**, have you had any of the following problems with your work or other regular daily activities **as a result of your physical health**? Yes/No
- Cut down the **amount of time** you spent on work or other activities
- **Accomplished less** than you would like
- Were limited in the **kind** of work or other activities
- Had **difficulty** performing the work or other activities (for example, it took extra effort)
- During the **past 4 weeks**, have you had any of the following problems with your work or other regular daily activities **as a result of any emotional problems** (such as feeling depressed or anxious)? Yes/No
- Cut down the **amount of time** you spent on work or other activities
- **Accomplished less** than you would like
- Didn't do work or other activities as **carefully** as usual
- During the **past 4 weeks**, to what extent has your physical health or emotional problems interfered with your normal social activities with family, friends, neighbors, or groups? Not at all/ Slightly/ Moderately/ Quite a bit/ Extremely
- How much **bodily** pain have you had during the **past 4 weeks**? None/ Very mild/ Mild/ Moderate/ Severe/ Very severe
- During the **past 4 weeks**, how much did **pain** interfere with your normal work (including both work outside the home and housework)? Not at all/ Slightly/ Moderately/ Quite a bit/ Extremely
- These questions are about how you feel and how things have been with you **during the past 4 weeks**. For each question, please give the one answer that comes closest to the way you have been feeling. How much of the time during the **past 4 weeks**... All of the time/ Most of the time/ A good bit of the time/ Some of the time/ A little of the time/ None of the time
- Did you feel full of pep?
- Have you been a very nervous person?
- Have you felt so down in the dumps that nothing could cheer you up?
- Have you felt calm and peaceful?
- Did you have a lot of energy?
- Have you felt downhearted and blue?
- Did you feel worn out?
- Have you been a happy person?
- Did you feel tired?
- During the **past 4 weeks**, how much of the time has **your physical health or emotional problems** interfered with your social activities (like visiting with friends, relatives, etc.)? All of the time/ Most of the time/ A good bit of the time/ Some of the time/ A little of the time/ None of the time
- How TRUE or FALSE is **each** of the following statements for you. Definitely true/ Mostly true/ Don’t know/ Mostly false/ Definitely false
- I seem to get sick a little easier than other people
- I am as healthy as anybody I know
- I expect my health to get worse
- My health is excellent

| **Supplementary table 1. Clinical characteristics in symptomatic men and women ≥65 years without diagnosed cardiac disease (n=17,069)** | | | | | | | | | | | | | | |
| --- | --- | --- | --- | --- | --- | --- | --- | --- | --- | --- | --- | --- | --- | --- |
| **Characteristics** | | | | | **Men** | | | | | **Women** | | | | |
|  | | | | | n=7,268 | | | | | n=9,801 | | | | |
|  | | | | |  | | | | |  | | | | |
| Mean age (years (SD)) | | | | | 73.7 ± 5.6 | | | | | 73.9 ± 5.4 | | | | |
| Median age (years (IQR) | | | | | 74 (69, 78) | | | | | 74 (69, 78) | | | | |
| Body mass index (mean, kg/m^2^ (SD)) | | | | | 27.2 ± 4.1 | | | | | 26.6 ± 4.7 | | | | |
| Obesity (body mass index ≥30 kg/m^2^) | | | | | 1,641/7,268 (22.6%) | | | | | 2,027/9,801 (20.7%) | | | | |
| Higher education, n | | | | | 3,743/7,267 (51.5%) | | | | | 4,808/9,796 (49.1%) | | | | |
| Employed, n | | | | | 1,165/7,268 (16.0%) | | | | | 759/9,801 (7.7%) | | | | |
| Living alone, n | | | | | 1,424/7,266 (19.6%) | | | | | 3,754/9,798 (38.3%) | | | | |
| Current smoker, n | | | | | 641/7,268 (8.8 %) | | | | | 739/9,801 (7.5 %) | | | | |
| Weekly alcohol use, n | | | | | 3,865/7,268 (53.2%) | | | | | 3,984/9,801 (40.7%) | | | | |
| Daily physical activity <30 min, n | | | | | 1,165/7,268 (16.0%) | | | | | 1,108/9,801 (11.3%) | | | | |
| Previous diseases | | | | |  | | | | |  | | | | |
| Diabetes mellitus, n | | | | | 1,243/7,157 (17.4%) | | | | | 1,068/9,663 (11.1%) | | | | |
| Hypertension, n | | | | | 5,069/7.050 (71.9%) | | | | | 6,792/9,540 (71.2%) | | | | |
| Stroke, n | | | | | 588/7,080 (8.3%) | | | | | 605/9,604 (6.3%) | | | | |
| Peripheral artery disease, n | | | | | 178/7,239 (2.5%) | | | | | 174/9,757 (1.8%) | | | | |
| Hypothyroidism, n | | | | | 354/7,124 (5.0%) | | | | | 1,750/9,691 (18.1%) | | | | |
| Hyperthyroidism, n | | | | | 56/7,140 (0.8%) | | | | | 233/9,706 (2.4%) | | | | |
| Chronic obstructive pulmonary disease, n | | | | | 704/7,009 (10.0%) | | | | | 662/9,481 (6.7%) | | | | |
| Obstructive sleep apnoea disorder, n | | | | | 1,210/6,319 (19.2%) | | | | | 893/8,650 (10.3%) | | | | |
| Anxiety, n | | | | | 936//6,954 (13.5%) | | | | | 1,615/9,275 (17.4%) | | | | |
| Medication | | | | |  | | | | |  | | | | |
| Platelet inhibitor, n | | | | | 1,868/71,74 (26.0%) | | | | | 1,840/9,742 (18.9%) | | | | |
| Lipid lowering therapy, n | | | | | 3,680/7,222 (51.0%) | | | | | 4,331/9,762 (44.4%) | | | | |
| Beta-blockers, n | | | | | 568/7,147 (8.0%) | | | | | 1,011/9,685 (10.4%) | | | | |
| Angiotensin-converting enzyme inhibitor, n | | | | | 519/7,145 (7.3%) | | | | | 523/9,694 (5.4%) | | | | |
| Angiotensin II receptor antagonist, n | | | | | 1,926/7,116 (27.1%) | | | | | 2,753/9,686 (28.4%) | | | | |
| **Supplementary table 2. Adjusted* odds ratio (OR) with 95% confidence interval for individual cardiac-related symptom among adults ≥65 years without diagnosed cardiac disease** | | | | | | | | | | | | | | |
|  | **Tachycardia** | | **Palpitations** | | | **Exertional dyspnoea** | | **Exertional chest pain** | | | **Syncope** | | **Fatigue** | |
| Female sex | 2.43 | (2.25-2.61) | 1.84 | (1.70-1.98) | | 1.31 | (1.22-1.41) | 0.86 | (0.73-1.00) | | 1.37 | (1.24-1.52) | 1.48 | (1.39-1.58) |
| Daily physical activity <30 min | 0.94 | (0.84-1.05) | 0.91 | (0.81-1.03) | | 1.73 | (1.57-1.90) | 1.19 | (0.96-1.48) | | 1.17 | (1.00 -1.35) | 1.59 | (1.46-1.74) |
| Current smoking | 1.12 | (0.98-1.28) | 0.97 | (0.84-1.12) | | 1.30 | (1.15-1.47) | 0.94 | (0.71-1.25) | | 1.02 | (0.84-1.23) | 1.31 | (1.17-1.47) |
| Obesity (body mass index ≥30 kg/m^2^) | 1.01 | (0.94-1.08) | 0.86 | (0.78-0.95) | | 2.01 | (1.86-2.18) | 1.22 | (1.01-1.46) | | 0.70 | (0.61-0.81) | 1.28 | (1.19-1.38) |
| Age <75 years | 1.50 | (1.39-1.62) | 1.38 | (1.28-1.50) | | 1.02 | (0.96-1.10) | 1.25 | (1.06-1.47) | | 0.82 | (0.74-0.91) | 1.07 | (1.00-1.14) |
| Living alone | 1.01 | (0.93-1.06) | 1.00 | (0.92-1.08) | | 1.24 | (1.15-1.33) | 1.00 | (0.84-1.18) | | 1.05 | (0.95-1.17) | 1.26 | (1.18-1.34) |
| Higher education | 1.01 | (0.92-1.11) | 1.16 | (1.08-1.24) | | 2.01 | (1.86-2.18) | 1.22 | (1.01-1.46) | | 0.70 | (0.61-0.81) | 1.28 | (1.19-1.38) |
| Employment | 1.05 | (0.95-1.17) | 0.92 | (0.82-1.03) | | 1.04 | (0.94-1.16) | 0.87 | (0.69-1.10) | | 1.01 | (0.86-1.18) | 0.95 | (0.86-1.04) |
| Weekly alcohol use | 1.04 | (0.97-1.12) | 0.99 | (0.92-1.06) | | 0.95 | (0.89-1.02) | 0.82 | (0.70-0.95) | | 1.04 | (0.94-1.14) | 0.76 | (0.72-0.81) |
| Comorbidity |  |  |  |  | |  |  |  |  | |  |  |  |  |
| Chronic obstructive pulmonary disease | 1.69 | (1.46-1.97) | 1.47 | (1.25-1.73) | | 15.11 | (13.19-17.31) | 2.37 | (1.84-3.04) | | 1.10 | (0.88-1.37) | 2.89 | (2.55-3.27) |
| Anxiety | 3.43 | (3.12-3.77) | 2.91 | (2.64-3.22) | | 2.05 | (1.85-2.26) | 2.38 | (1.97-2.88) | | 1.98 | (1.74-2.27) | 4.30 | (3.93-4.69) |
| Hyperthyroidism | 1.62 | (1.26-2.10) | 1.39 | (1.04-1.83) | | 1.72 | (1.33-2.22) | 1.42 | (0.82-2.46) | | 1.65 | (1.18 -2.31) | 1.79 | (1.42-2.26) |
| Obstructive sleep apnoea disorder | 1.31 | (1.18-1.46) | 1.34 | (1.20-1.50) | | 1.67 | (1.51-1.84) | 1.80 | (1.48-2.18) | | 1.03 | (0.88-1.20) | 2.17 | (1.99-2.37) |
| Stroke | 1.18 | (1.03-1.35) | 1.04 | (0.91-1.20) | | 1.43 | (1.26-1.62) | 1.40 | (1.08-1.81) | | 1.38 | (1.16-1.64) | 1.68 | (1.51-1.88) |
| Peripheral artery disease | 1.13 | (0.87-1.46) | 1.02 | (0.77-1.35) | | 1.53 | (1.22-1.93) | 1.58 | (1.01-2.49) | | 1.21 | (0.86-1.70) | 1.44 | (1.17-1.77) |
| Hypothyroidism | 1.05 | (0.94-1.17) | 1.15 | (1.03-1.84) | | 1.17 | (1.05-1.30) | 1.56 | (1.26-1.93) | | 1.11 | (0.96-1.28) | 1.46 | (1.33-1.60) |
| Hypertension | 1.33 | (1.23-1.45) | 1.26 | (1.16-1.38) | | 1.29 | (1.19-1.39) | 0.85 | (0.72-1.00) | | 1.03 | (0.93-1.15) | 1.13 | (1.06-1.21) |
| Diabetes mellitus | 0.92 | (0.83-1.03) | 0.91 | (0.82-1.01) | | 0.99 | (0.90-1.09) | 1.04 | (0.84 -1.27) | | 1.00 | (0.86-1.149) | 1.37 | (1.27-1.49) |

*Adjusted for all listed factors
